# Digital replica to unveil the impact of growing conditions on orange postharvest quality

**DOI:** 10.1038/s41598-024-65285-w

**Published:** 2024-06-23

**Authors:** Daniel Onwude, Paul Cronje, Jade North, Thijs Defraeye

**Affiliations:** 1 Laboratory for Biomimetic Membranes and Textiles, Empa, Swiss Federal Laboratories for Materials Science and Technology, Lerchenfeldstrasse 5, CH-9014 St. Gallen, Switzerland; 2 Citrus Research International, Department of Horticultural Science, University of Stellenbosch, Private Bag X1, Stellenbosch, 7602 South Africa; 3 Food Quality and Design, Wageningen University & Research, P.O. Box 17, 6700 AA Wageningen, the Netherlands

**Keywords:** Digital twin, Climate variability, Horticulture, Citrus fruit, Cold chain, Food waste, Chemical engineering, Nutrition, Computational models, Climate and Earth system modelling, Climate-change impacts

## Abstract

The postharvest end-quality of citrus is significantly impacted by pre-harvest factors such as weather, which varies among growing regions. Despite the importance of these factors, the influence of regional weather variations, such as variations in temperature, humidity, wind, vapor pressure deficit (VPD), and solar radiation on postharvest citrus quality, is largely unknown. This study aims to quantify this impact through a physics-driven digital replica of the entire value chain of Valencia oranges, from orchards in South Africa to retail in Europe. Predicted fruit properties data at harvest and hygrothermal sensor data from orchard to retail for different production regions are coupled to a physics-based fruit model to simulate key postharvest fruit quality metrics. These metrics include mass loss, chilling injury, fruit quality index (FQI), remaining shelf life (RSL), total soluble solids (TSS), and titratable acidity (TA). Our digital fruit model reveals that regional weather variability significantly affects fruit quality evolution when comparing data from Nelspruit, Letsitele, and Sunday’s River Valley (SRV). The impact of weather variations is most pronounced in the temperate oceanic climate of SRV compared to the hotter climates of Letsitele and Nelspruit. Our findings indicate that differences in weather conditions between these growing regions impact postharvest mass loss, FQI, RSL, TSS, and TA of Valencia oranges at retail. The impact is up to 10% variation in mass loss and RSL, 4% in TSS, and 1% in TA among oranges grown in different regions. We show that temperature and humidity variations in the postharvest local transport of oranges between different regions largely increase mass loss by up to twofold, FQI by up to ~ 12%, and RSL by up to ~ 15% at retail. Our research also shows that weather temperature is the most important metric during fruit growth affecting various aspects of postharvest orange quality. This study offers valuable insights into the impact of regional weather variations on the quality of oranges available to consumers. These findings could help the citrus industry enhance growing practices, postharvest logistics, retail marketing, and cold chain strategies, thereby improving product quality and consumer satisfaction.

## Introduction

Citrus is an extensively grown fruit crop worldwide, mainly cultivated in tropical and sub-tropical climates^1^. Its reputation is due to its exceptional ability to quench thirst, refreshing fragrance, and abundant vitamin C^2,3^. Citrus and its products are also a rich source of minerals and dietary fibre essential for overall human nutritional well-being. For example, oranges generally contain 40–130 mg of vitamin C/100 g, which is within the recommended daily intake (RDI) of vitamin C for humans^4–7^. In addition, citrus peels contain bioactive flavonoids with antioxidant, anti-inflammatory, and potential anticancer properties, which offer various human health benefits^8–11^. Despite their high nutritional value, citrus are susceptible to varying quality losses during their postharvest journey, one of which is due to the differences in their growing conditions, among others^12–14^.

Several pre-harvest factors, including maturity stage^15^, rootstock type and selection^16^, alternative bearing and fruit drop^17^, metabolic status^18^, soil quality^19^, cultivar selection^19^, horticultural practices^20^, and weather conditions^19^ affect the postharvest quality and shelf life of citrus. While researchers have investigated extensively how most of these factors influence various aspects of postharvest fruit quality, including nutritional value, and shelf life, the impact of weather variation is largely unknown. This is surprising, considering that the environmental weather conditions of a citrus growing region play a critical role in determining citrus growth and development. Weather conditions, such as temperature, humidity, rainfall, radiation, wind speed, and light exposure, vary significantly between citrus growing regions^21^. These variations lead to significant differences in the physicochemical properties of fruit at harvest, including fruit size, rind thickness, acidity, and sugar content^21,22^. These initial biological differences at the start of the citrus postharvest journey could result in significant variation in the postharvest end quality, such as remaining shelf life, mass loss, overall fruit quality index, and sugar content^14^. As such, the prevailing weather conditions during the different stages of fruit growth and development directly impact the postharvest end-quality of citrus.

Despite this critical impact, few existing studies have quantified it, with research focusing on the effect of temperature, humidity, precipitation, rainfall, wind, and solar radiation on fruit quality at harvest^22–28^. However, growing weather conditions affect not only the quality and yield of fruit at harvest but also play a crucial role in extending postharvest shelf life, especially due to its impact on the temperature gradient of fruit at harvest^21^. Additionally, few studies have shown that rainfall patterns, for instance, significantly affect the shelf life of edible plants such as tomato^29,30^ and pomegranate^31,32^ during storage. Rarely did researchers investigate the effect of multiple weather parameters on fruit quality along an actual supply chain. To the best of our knowledge, no study has assessed the impact of weather variability (so differences in weather parameters such as temperature, humidity, etc.) between citrus-producing regions on the postharvest quality of fruit at retail within an actual global maritime citrus cold chain. To this end, the relevant research questions are: (1) How do different pre-harvest weather conditions impact the initial quality of citrus fruit at harvest? (2) What is the impact of initial quality variability, resulting from differing pre-harvest weather conditions, on the postharvest quality of citrus fruit at retail within a global maritime cold chain? (3) Which weather parameter (e.g. temperature, humidity, and rainfall) has the largest impact on the final quality of citrus fruit delivered to consumers? These questions are critical to understanding and quantifying the influence of growing conditions on citrus postharvest end quality and to determine the key factors that affect fruit quality during its journey from orchard to consumer.

The global maritime citrus cold chain is marked by fluctuations in hygrothermal and ventilation conditions from the orchard to the consumer. As such, it is essential to capture the physical processes influencing the quality of the fruit throughout the entire supply chain. Physics-based digital twins have recently been developed to unveil fruit quality evolution and assess the impact of postharvest hygrothermal variability throughout the entire supply chain^14,33,34^. These digital twins, also known as digital replicas, employ mechanistic simulations to mimic essential fruit characteristics and relevant physiological, metabolic, and hygrothermal processes^14,35^. These simulations are linked to real-world environmental conditions through real-time sensor data. By providing real-time quality predictions, these digital twins can improve supply chain efficiency and enable stakeholders to quickly adapt to changing market demands, ensuring consumers consistently receive high-quality citrus. For instance, these virtual models could empower various actors within the citrus supply chain, including suppliers and retailers, to make informed decisions about when and from which growing regions to source their supply. However, such a digital twin model does not currently exist.

This study aims to predict the impact of weather differences–temperature, humidity, rainfall, and vapor pressure deficit, between citrus-growing regions on the postharvest quality and shelf life of citrus at retail via a physics-inspired digital twin. We developed a mechanistic virtual model of oranges, covering their journey from orchard to retail for each growing region. By coupling measured weather parameters during growth and development—Such as temperature, humidity, wind speed and radiation, for each growing region with a mechanistic plant growth model, we predict the physicochemical properties of fruit for each region. These varying initial fruit properties per region and hygrothermal sensor data from orchard to retail are fed into a physics-based fruit model to simulate the fruit's evolution until it reaches the consumer. Our digital twin approach allows us to assess the impact of regional weather variability on actionable postharvest quality metrics of oranges. These actionable metrics include mass loss, low-temperature-induced chilling injury, fruit quality index (FQI), remaining shelf life (RSL), total soluble solids (TSS), and total titratable acidity (TA). Through this mechanistic-driven simulation, we directly quantify the impact of regional weather variability on the postharvest quality of oranges within an idealized citrus maritime supply chain (so considering the local unrefrigerated supply chain from the orchard to pre-cooling). Additionally, we determine the impact of variability in growing conditions on the postharvest quality of oranges based on actual citrus maritime supply chain scenarios. This digital replica approach enhances traditional physics-based modeling, offering comprehensive end-to-end coverage, enabling stage-specific analysis, and delivering actionable insights. These lead to more accurate predictions and better decision-making regarding the postharvest end quality of citrus fruit. Note that this study did not consider feedback from the digital twin.

## Materials and methods

### Study area

In this study, we focused on ‘Turkey’ Valencia oranges that were produced during the 2018/2019 season in the main citrus-growing regions of South Africa (SA). Three major citrus growing regions of South Africa (SA), with distinctly different climatic zones, were included. These areas are the hot semi-arid climate of Letsitele in Limpopo, the humid subtropical climate of Nelspruit in Mpumalanga, and the temperate oceanic climate of Sunday's River Valley (SRV) in Eastern Cape. For more information about the production details of the experimental sites, we refer readers to our previous researched work^36^.

#### Weather data

Weather data for various regions during the 2018/2019 citrus production season, spanning the entire fruit development period, were acquired through the use of automated weather stations operated by the Agricultural Research Council (ARC). This dataset includes daily measurements of minimum and maximum temperature (in °C), minimum and maximum relative humidity (expressed as % RH), and recorded rainfall (in millimeters). Furthermore, we computed the vapor pressure deficit (VPD). This metric quantifies the difference between the actual moisture content in the air and the maximum moisture it can hold when saturated at a specific temperature. We also collected data from NASA for wind speed (U_speed_, m s ^−1^), short-wave (W m ^−2^), and long-wave (W m ^−2^) radiation during the growth process for Letsitele, Nelspruit, and Sunday's River Valley (SRV), via https://power.larc.nasa.gov/data-access-viewer/. These weather parameters were used to predict the fruit physicochemical properties at harvest for the three citrus production regions of South Africa. For this study, the modeled physicochemical properties considered are fruit weight (FW), fruit size (FD), rind weight (RW), rind thickness (RT), total soluble solids (TSS), titratable acidity (TA), thermal conductivity of orange pulp (k_pulp_), thermal conductivity of orange rind (k_rind_), specific heat capacity of the orange (C_p,fruit_), and initial fruit temperature (T_,fruit,ini_). Please refer to our previous research paper for more comprehensive information on the weather datasets and how they were used in predicting the physicochemical characteristics of oranges in different regions at harvest^21^.

#### Semi-mechanistic pre-harvest digital twin

We developed semi-mechanistic pre-harvest digital twins of 50 Valencia oranges per growing region, representing an average of 15 fruit per tree from 10 trees across 5 different orchards per growing region for the 2018/2019 citrus production seasons. These digital twins integrate a crop growth model, regression models, and a thermal model for heat stress with real-world pre-harvest climate data such as daily average temperature, relative humidity, heat units, wind speed, and solar radiation to predict fruit quality at harvest.

For the crop growth model, we used the EPIC crop model, primarily based on a collection of empirical functions for various plant growth processes and potential of the growth process^37^. Potential fruit growth depends on weather variables such as daily maximum and minimum temperatures and the heat units from the previous day. The fruit growth process is, therefore, driven by the daily heat unit. Based on the effective heat unit values, we calculated the heat unit index (HUI), which ranges from 0 at the start of the fruit set to 1 at physiological maturity (harvest time), and also computed the heat unit factor (HUF).

We used the relationship between heat units, solar radiation, wind speed, relative humidity, air temperature, soil temperature, maximum fruit weight, and the minimum crop stress factor (REG) to predict the growth of a single Valencia orange. The EPIC model outputs the predicted fruit weight for each tree in each orchard within citrus-producing regions of South Africa. Additionally, we developed empirical regression functions and coupled them to the EPIC model to predict the fruit diameter (mm), rind weight (g), rind thickness (mm), total soluble solids (°Brix TSS), and titratable acidity (% TA) during growth and development and also at harvest. Following the approach of Saudreau et al.^38^ and Saudreau et al.^39^, we solved for the spatial and temporal variations in heat load (thermal stress) on the fruit surface due to its interaction with the environment during growth to predict the thermal properties and initial fruit temperature at harvest. Details and comprehensive explanations are available in our previous study^21^. Therefore, only the final predicted physicochemical properties of Valencia oranges at harvest for Letsitele, Nelspruit, and Sunday's River Valley (SRV) of South Africa were used in this study.

#### Maritime cold chain of oranges from SA to Western Europe

Once oranges are harvested, they are transported to packhouses by unrefrigerated trucks. At the packhouse, they undergo cleaning, degreening (early harvested fruit), sorting, ozone and chemical treatments, waxing, grading, quality checks for defects, packaging, and storage. Thereafter, fruit are loaded onto unrefrigerated distribution trucks or trains (depending on the packhouse location) and transferred to the cold rooms. The distance between the closest packhouse and a cold store could be 200 km. The entire process, from harvesting to pre-cooling, can take 48–72 h.

The cold rooms are often located close to the shipping port. The pallets of oranges are precooled from about 12–20 °C to 4 °C within 24–36 h. Afterward, the pallets are transferred to a storage room (at 2 °C) or loaded into containers with Genset and transported by trucks, depending on when they are transported to the container vessel at the port. The set temperature of the refrigerated trucks ranges from − 1.5 to 1 °C.

### Experimental measurement

#### Fruit quality parameters at harvest

We previously developed mechanistic and empirical models to predict the growth and harvest quality of Valencia oranges produced in different regions of South Africa. To validate our models, we compared them with experimental data obtained from ‘Turkey’ Valencia oranges harvested from five commercial orchards in each of the three major growing regions in South Africa for the 2018/2019 growing season. A total of 150 fruit per orchard were harvested by selecting 15 fruit from the outside up to 30 cm into the canopy. Our experimental data showed that the predicted quality parameters at harvest matched well with the observed values. Readers can refer to our previous research work for more details about the experimental measurements and model validation^40^. Therefore, only relevant information is included in this paper.

#### Measurement of air temperature in actual cold chain

For the postharvest local supply chain of harvested oranges from orchards to cold storage in South Africa, environmental temperature and humidity data during transport were obtained from NASA, via https://power.larc.nasa.gov/data-access-viewer/. This was done for Nelspruit, Letsitele, and Sunday's River Valley (SRV) in South Africa during the citrus harvest period of 15–26 August 2019 based on the available shipment data. 

The air temperature on the cold chain of oranges was monitored and data for a single shipment from the cold store in Durban, South Africa, to a distribution center (DC) in Rotterdam, Netherlands, were acquired. This data, which represents a realistic temperature–time profile for overseas cold chain, was collected using TempTale®4 (TT4) GEO Eagle Extended (SENSITECH, Beverly, MA, USA) sensors with an accuracy of ± 0.5 °C. The air temperature set point was − 1.5 °C. More details about the sensor positioning and approach for data acquisition in the cold citrus chain are available in our earlier published study^14^.

We also extended the hygrothermal monitoring from the DC to retail stores, assuming three days of retail sales conditions. We do this using stochastic simulation of the average air temperature (°C) at a retail location in Rotterdam, Netherlands. Data was collected via NASA POWER (https://power.larc.nasa.gov/data-access-viewer/). More details can be found in Ref.^14^.

**Figure 1:**
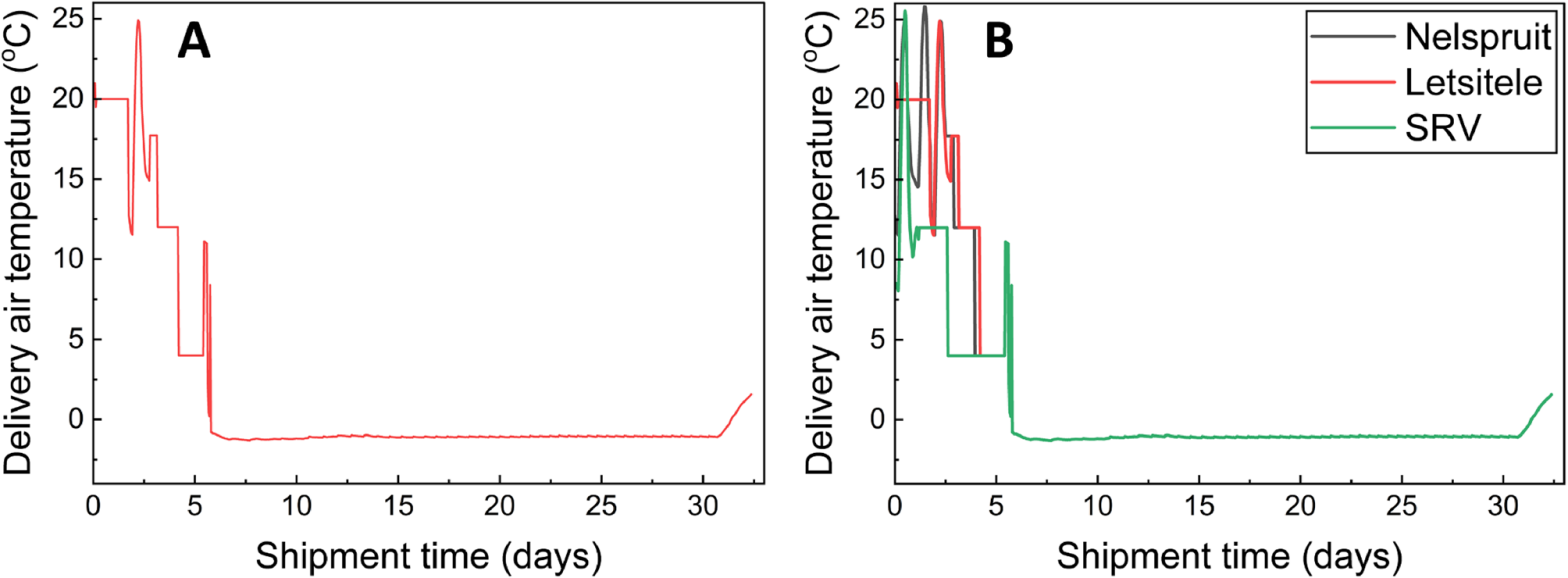
Time–temperature profile of transcontinental cold chain of citrus (− 1.5 °C targeted delivery cold air temperature and shipment length of 32 days) from Farms in South Africa (SA) to a retail shop in Europe (EU). (**A**) for shipment for Letsitele used for scenario 1, (**B**) for shipments from Nelspruit, Letsitele, and SRV for scenario 2.

The hygrothermal data collection was done in two different scenarios. *Scenario 1* assumes that the oranges harvested from Letsitele, Nelspruit and SRV go through the same local and transcontinental cold chain. This scenario is not ideal as, in reality, there is variability in the local citrus transport chain from the farm to cold storage and from cold storage to the retailer. However, this scenario will enable us to truly test climate variability's effect on long-term cold transport and storage. *Scenario 2* considers the unrefrigerated local citrus transport variability between oranges collected from orchards in Letsitele, Nelspruit and SRV until cold storage in Durban, South Africa. This scenario is typical of the actual commercial maritime citrus cold chain. We have already reported the findings of the effect of cold chain variability on end-quality of citrus^14^. Therefore, we assumed the same chain length for oranges from all growing regions in this study. As a result, the fruit harvested from regions close to the cold store (e.g. SRV) stayed slightly longer in the cold store than those from farther areas (e.g. Letsitele). Please see Sect. “Maritime cold chain of oranges from SA to Western Europe” for describing a typical transcontinental cold chain of citrus. Figure 1 illustrates the temperature–time history of the transcontinental cold chain of citrus for Scenario 1 and Scenario 2.

### Physics-based digital twin configuration of a single fruit

**Figure 2:**
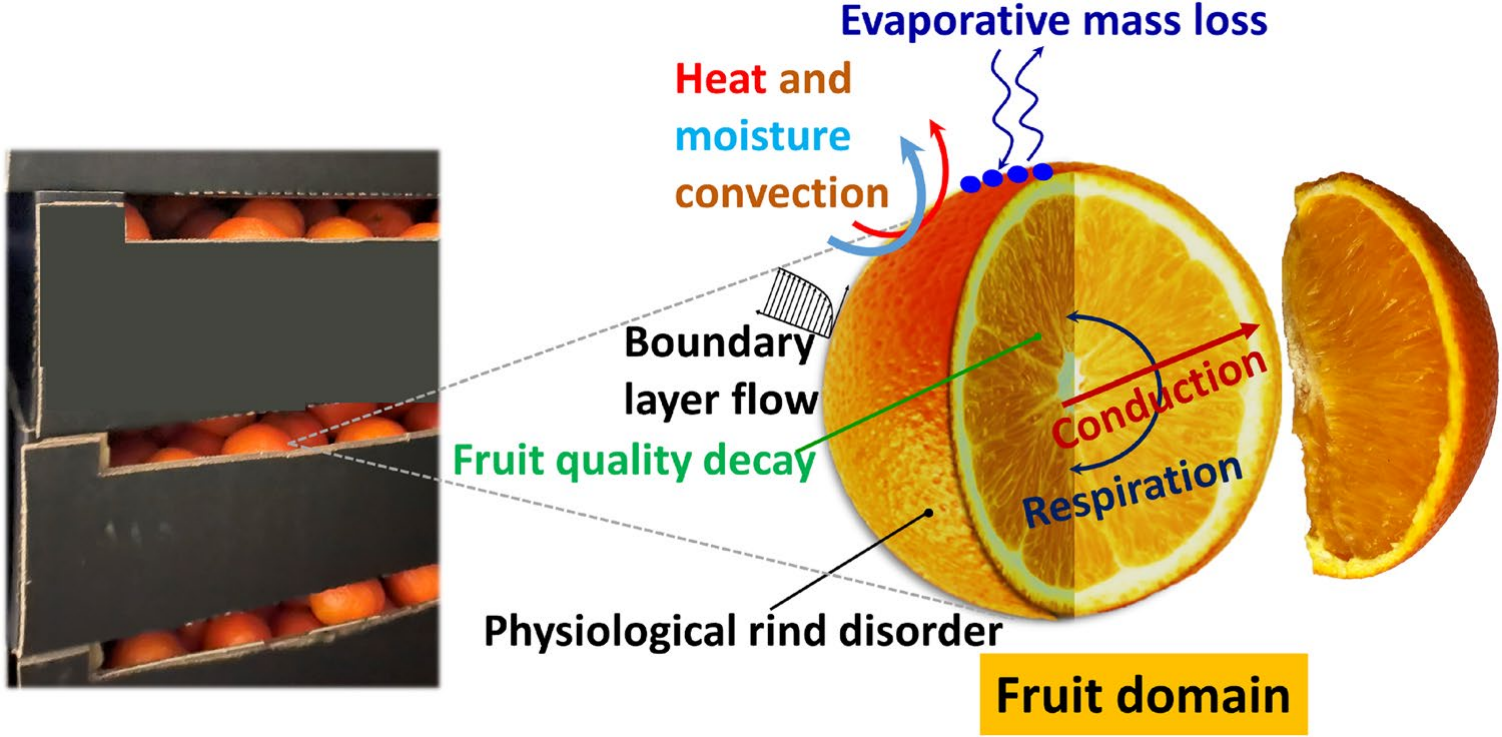
Geometry and boundary conditions of orange during postharvest transcontinental cold chain (figure not to scale).

We created postharvest physics-based digital twins of Valencia orange to quantify the impact of weather differences between citrus growing regions on the postharvest fruit quality. To accomplish this, we modeled a single fruit as a two-dimensional axisymmetric geometry of a sphere, dividing the fruit into two sections—the rind and the fruit pulp (Fig. 2). The domain was divided into two sections of the fruit – the rind (thickness ((rind_thick_) = 5.9 mm) and the fruit pulp (pulp radius (r_pulp_) = 30.7 mm). Our digital twin simulated the hygrothermal and physiological processes during the cold chain from orchards to fork, mimicking the actual maritime supply chain of citrus with a single shipment. To develop our model, we used a physics-based mechanistic approach based on the finite element method and linked this to measured air temperature data around the fruit vicinity. We collected hygrothermal data from orchards in South Africa to the retail market in Western Europe, and forecasted how several ongoing processes manifest in the actionable final product quality metrics such as mass loss, fruit quality index (FQI), remaining shelf life (RSL), total soluble solids (TSS, °Brix), titratable acidity (TA, %), and chilling injury (%). We used the output fruit parameters of our pre-harvest digital twin for Letsitele, Nelspruit, and SRV^40^ as initial conditions. These parameters include FW, FD, RW, RT, TSS, TA, thermal conductivity of orange pulp, thermal conductivity of orange rind, specific heat capacity of the orange, and average fruit temperature, with varying values for different growing regions. The pre-harvest digital twin was developed based on the weather differences between different growing regions using EPIC crop model and regression models.

### Mechanistic multiphysics model for postharvest supply chain

We used our previously developed mechanistic postharvest model^14^ to extend the pre-harvest digital twin to the end of the postharvest cold chain. This was done by simulating orange fruit's heat and mass transfer process throughout the supply chain from farm to retail. We also coupled already developed and validated physics-based quality models, including mass loss (%), overall fruit quality or fruit quality index (FQI, %), remaining shelf life (days), total soluble solids (TSS, °Brix), titratable acidity (TA, %), and chilling injury (%) models with the heat transfer model^14^. Therefore, only the main model features and equations are highlighted here. The main model assumptions are:

- Thermal equilibrium between all components and phases was assumed in this model.
- No radiation exchange between fruit.
- Forced convection was dominant during precooling and transport, so there was no buoyancy effect.
- The initial quality of oranges after harvest from all growing areas is 100%.
- The quality threshold value of 10% was assumed as an acceptable limit for consumers.
- A mass loss threshold of 10% was also assumed for oranges during shipment^41^.

#### Thermal model

The energy conservation equation was solved in the fruit, as given below:

1 $${\rho }_{i}{C}_{p,i}\frac{{\delta T}_{i}}{\delta t}+\nabla \cdot \left(-{k}_{i}\nabla {T}_{i}\right)={Q}_{resp,i}.$$

*ρ*_i_ is the density (kg m^–3^), *C*_p,i_ is the specific heat capacity (J kg^–1^ K^–1^), ${T}_{i}$ is the fruit temperature (K), ${k}_{i}$ is the material's thermal conductivity (W m^–1^ K^–1^), $\nabla$ is the spatial derivative operator, and *i* represents the fruit pulp and rind domain. ${Q}_{resp,i} (\text{W }{m}^{-3})$ represents the volumetric heat of respiration during plant growth.

The temperature-driven convective heat transfer flux was calculated based on flux continuity as presented:

2 $$\mathbf{n}.\left(k\nabla T\right)= {H}_{c}\left({T}_{air}-{T}_{rind}\right)-{j}_{m}.{E}_{vap},$$

where **n** is the unit vector normal to the surface (–), *H*_c_ is the convective heat transfer coefficient (W m^–2^ K^–1^), *T*_air_ is the delivery air temperature (K), *j*_m_ is the moisture flux at the surface (kg m^–2^ s^–1^) derived from the moisture transport model and *E*_vap_ is the latent heat of evaporation^42^.

We accounted for the hygrothermal behavior of the fruit during cooling using the convective heat and mass transfer coefficients (*H*_c_, *k*_air_). We estimated the actual airspeed in the fruit box during oversea container storage and transport using the following equation:

3 $${U}_{actual}= \frac{{U}_{superficial}}{\varnothing }=\frac{{Q}_{air}}{{A}_{cargo} x \varnothing },$$

where ${U}_{actual}$, (= 0.108 m s^–1^) is the actual airspeed around the orange fruit, ${U}_{superficial}$, (0.042 m s^–1^) is the superficial airspeed,${Q}_{air}$ (= 4000 m^3^ h^–1^) is the delivery air flow rate, ${A}_{cargo}$, (= 26.45 m^2^) is the cross-sectional area of the cargo bottom, and *ϕ* (0.39) is the porosity of an orange pallet. In addition to cold storage and transport, we also assumed ${U}_{actual}$(= 0.108 m s^–1^) to be the airspeed around the orange during precooling and transport to the retail store from DC. From the farm in South Africa to pre-cooling, we assumed an actual airspeed was 2 m s^−1 ^based on data from NASA.

The dependency of the spatially-varying heat transfer coefficient (*H*_c_, W m^–2^ K^–1^) on airspeed is explained by the Nusselt number (*Nu*) correlation for flow around a spherical shaped object^43^:

4 $$Nu=\frac{{H}_{c} \text{FD}}{{k}_{air}}=2+ \left(0.4{Re}^{0.5}+0.06{Re}^{0.667}\right){Pr}^{0.4}{\left(\frac{{\mu }_{air}}{{\mu }_{air,rind}}\right)}^{0.25}.$$

Here, *Re* (= $\text{FD} {U}_{actual}$/${\upsilon }_{air}$). Note that ${H}_{c}$ values differ per tree, per orchard and per growing area as a result of changing fruit dimension (FD).

#### Mass loss model

The mass flux (*j*_m_) at the fruit surface (kg m^–2^ s^–1^), was calculated based on the following equations^44,45^.

5 $${\dot{j}}_{m}= {k}_{t} \left({P}_{v,rind}-{P}_{v,air}\right),$$

where ${k}_{t}$ is the convective mass transfer coefficient (s m^–1^), ${P}_{v,rind},$ is the vapor pressure on the fruit surface (Pa) and ${P}_{v,air}$ is the ambient vapor pressure (Pa).

We calculated ${k}_{t}$ from the equation below:

6 $${k}_{t}={\left(\frac{1}{{k}_{air,mass}}+\frac{1}{{k}_{rind}}\right)}^{-1}.$$

The air film mass transfer coefficient ($${k}_{air,mass}$$) was estimated based on the relationship between the coefficient of diffusion of water vapor in air (*δ*_wv,air_, 2.19 × 10^–05^ m^2^ s^–1^), Schmidt number (Sc, 0.67) and Sherwood number (*Sh*, depends on FD) for a sphere. $${k}_{rind}$$ (= 1.72 × 10^–9^, s m^–1^) represents the moisture transport resistance to the fruit rind. The vapor pressures where obtained by dynamically linking the fruit surface temperature ($${T}_{rind}$$ for $${P}_{v,rind})$$ and the air temperature ($${T}_{air}$$ for $${P}_{v,air})$$ using the Antoine equation^44^. Our previously published work shows the calculation steps, model calibration, and validation^14^.

We modeled the transpiration-driven mass loss (*ML*, %) from the equation below^14^:

7 $$ML=\frac{\Delta {j}_{m} . {A}_{s}}{{FW}_{ini}} \times 100,$$

where $\Delta {j}_{m}$ is the integral of mass flux over time, *A*_s_ is the surface area of the fruit (m^2^) and ${FW}_{ini}$ is the initial weight of the fruit after harvest (kg), which varies per tree, orchard and growing area.

#### Kinetic model for fruit quality attributes

In this study, kinetic models were implemented to quantify the evolution of fruit quality index (FQI, %), remaining shelf life (days), total soluble solids (TSS, °Brix) and titratable acidity (TA, %), as in the equation below:

8 $$\frac{-d{A}_{i}}{{d}_{t}}= {k}_{i}{A}_{i}^{{n}_{i}},$$

And,

9 $${k}_{i}\left(T\right)= {K}_{0,i}{e}^{\frac{{-E}_{a,i}}{RT}},$$

where *A*_*i*_ is the respective quality attribute, subscript *i* represent the specific attribute, *k*_*i*_ is the rate constant (*s*^−1^), *n*_*i*_ is the reaction order (1st order for TSS, TA, and FQI), k_0,i_ is a constant (= 148 for FQI, − 1 for TSS, 5925 for TA, s^–1^), E_a,i_ represents the activation energy (= 45,229, for FQI, 49,524 for TSS, 60,218 for TA, J mol^–1^), T is the absolute temperature (K), and R is the ideal gas constant (8.314 J mol^–1^ K^–1^). The calibration of the different quality models has already been reported in our previous work^14^, except for TA.

The titratable acidity (TA, %) model was calibrated based on storage experiment in the literature for 'Valencia' oranges^46^. Valencia orange can be stored between 7 and 28 days at 20–5 °C. The initial value was 1.24% at the start of the shipment and final value after storage was 1.16%^46^. To account for the dependence of TA evolution on storage temperature, we assumed a *Q*_10_ factor of 2. *Q*_10_ is typically about 2 to 3 for degradation reactions in fruit^47,48^. A *Q*_10_ of 2 means that the shelf life of the fruit is halved for every 10 °C rise in storage temperature, so the fruit can be stored for 56 days at 0 °C, 28 days at 10 °C, and 14 days at 20 °C.

10 $${Q}_{10}= \frac{{k}_{T+10}}{{k}_{T}},$$

which then can be rewritten as follows^49^.

11 $${Q}_{10}=\frac{{k}_{0, i}{e}^{\frac{- {E}_{a,i}}{R(T+10)}}}{{k}_{0,i}{e}^{\frac{-{E}_{a,i}}{RT}}}= \frac{{e}^{\frac{-{E}_{a,i}}{R(T+10)}}}{{e}^{\frac{-{E}_{a,i}}{RT}}}={e}^{-{E}_{a,i(}\frac{1}{R\left(T+10\right)}-\frac{1}{RT})},$$

12 $$\text{ln}{Q}_{10}=\frac{10{E}_{a,i}}{RT(T+10)}\approx \frac{10{E}_{a,i}}{R{T}^{2}}.$$

13 $${E}_{a,i}\approx \frac{R{T}^{2}}{10}\text{ln}{Q}_{10}.$$

With this E_a_,_i_ value, k_0,i_ was calculated by rearranging Eqs. (9) and (13) as:

14 $${k}_{0,i} = \frac{{k}_{i}(T)}{{e}^{\frac{-{E}_{a,i}}{RT}}}=\frac{- \frac{1}{t} \text{ln}(\frac{{A}_{i} \left(t\right)-C}{{A}_{0,i}})}{{e}^{\frac{-{E}_{a,i}}{RT}}},$$

where *k*_(T+10)_, *k*_T_ correspond to the rate constants at temperatures (*T* + *10*) and *T* [K], respectively. ${A}_{0,i}$ is the initial TA value (1.24%, at t = 0 s), E_a,i_ is the activation energy [J mol^–1^], R is the ideal gas constant (8.314 J mol^–1^ K^–1^) and C is an integration constant. Note that the remaining shelf life (RSL) was modelled using the kinetic rate model by storing the oranges at retail conditions of 20 °C. The initial values for TSS, and TA varied per tree, orchard and growing region and are presented in Table 1.

**Table 1:** Parameters and average initial values for orange fruit harvested at different growing regions based on pre-harvest model predicted output at harvest.

Parameters	Symbol	Units	Letsitele	Nelspruit	SRV
Fruit weight	FW	(g)	233.27	226.26	150.18
Fruit diameter	FD	(mm)	80.06	79.38	67.30
Rind thickness	RT	(mm)	4.44	4.50	4.99
Rind weight	RW	(g)	101.28	97.41	85.09
Total soluble solids	TSS	(°Brix)	12.26	12.83	12.35
Titratable acidity	TA	(%)	1.29	1.02	2.19
Specific heat capacity of fruit	${C}_{p,fruit}$	(J kg^–1^ K^-1^)	4325.90	4332.99	4273.00
Thermal conductivity of pulp	${k}_{pulp}$	(W m^–1^ K^–1^)	0.56	0.56	0.55
Thermal conductivity of rind	${k}_{rind}$	(W m^–1^ K^–1^)	0.51	0.51	0.50
Initial fruit temperature	T,_fruit,ini_	(^o^C)	14.34	16.85	15.08

#### Thermal-driven model for chilling injury

The chilling injury severity on fruit rind was modeled in a similar manner to the thermal damage model for the human skin during skin burn^50^. The model quantifies thermal damage as a dimensionless damage integral (Ω) based on the combined effect of rind temperature (*T*_rind,_) (K) and exposure time (*t*):

15 $$\Omega \left(\text{t}\right)=\underset{0}{\overset{t}{\int }}{k}_{0,ci}.{e}^{\left(-\frac{{E}_{a,ci}}{R.{T}_{rind}}\right)}.dt,$$

where *k*_0,ci_ is the pre-exponential factor (s^–1^), and *E*_a,ci_ is the activation energy (J∙mol^–1^). The model description and calibration steps are available here^14^.

### Numerical simulation

Our physics-based simulation was implemented in COMSOL Multiphysics (version 6.1), a commercial finite element-based software, in which we used the same time-dependent solver configuration as in our previous study^14^. We solve the heat transfer and thermal damage in the fruit during convective cooling using the ‘Bioheat Transfer' physics. We specify different material properties per climate zone for the damaged tissue for the thermal damage model. So, this considers the variations in the physiochemical properties of fruit at harvest between growing regions. We further use the ODE module to solve for mass loss, FQI, RSL, TSS and TA. After grid sensitivity analysis^14^, we used triangular and quadrilateral finite elements, with a total average element size ranging from 6630 to 6704, depending on the growing area. We used parametric sweeps of transient studies to capture the variability of oranges from 150 trees in five orchards within a growing area. The simulation used a quadratic Lagrange geometry shape function and a MUMPS (MUltifrontal Massively Parallel sparse direct Solver) fully-coupled direct Solver. The solver tolerance was set to 10^–5^ and a simulation time step of 3600 s was adopted, corresponding to the time interval of the hygrothermal shipment data collected. The internal time steps to reach convergence were taken smaller and set by the solver tolerance. Table 1 presents the average initial values for fruit properties across the growing regions.

### Statistical data analysis

To assess the impact of variability between growing regions on the physicochemical properties of fruit at harvest, a one-way analysis of variance (ANOVA) using Fisher tests was performed for each parameter. To investigate the impacts of regional weather variability on the quality of oranges at the end of their postharvest life, a one-way analysis of variance (ANOVA) was performed. Digital twin output for different growing regions was presented as median (centre line), 75th upper and 25th lower quartiles (box limits), and 1.5 × the interquartile range (IQR, whiskers) with a 0.95 confidence level. We used Fisher LSD test at p ≤ 0.05 significant level and 95% confidence interval to assess if there were significant differences in the fruit quality attributes within groups. Pearson's correlation coefficients (Pearson's r) at p = 0.05, 0.01, and 0.001 significant levels were used to statistically determine the correlation between the fruit quality attributes and weather parameters. All analyses were conducted using ORIGINPRO 2022 (64-bit) SR1 (Government) (OriginLab, Northampton, Massachusetts, USA).

## Results and discussion

### What is the impact of variability between growing regions on the physicochemical properties of fruit at harvest?

**Figure 3:**
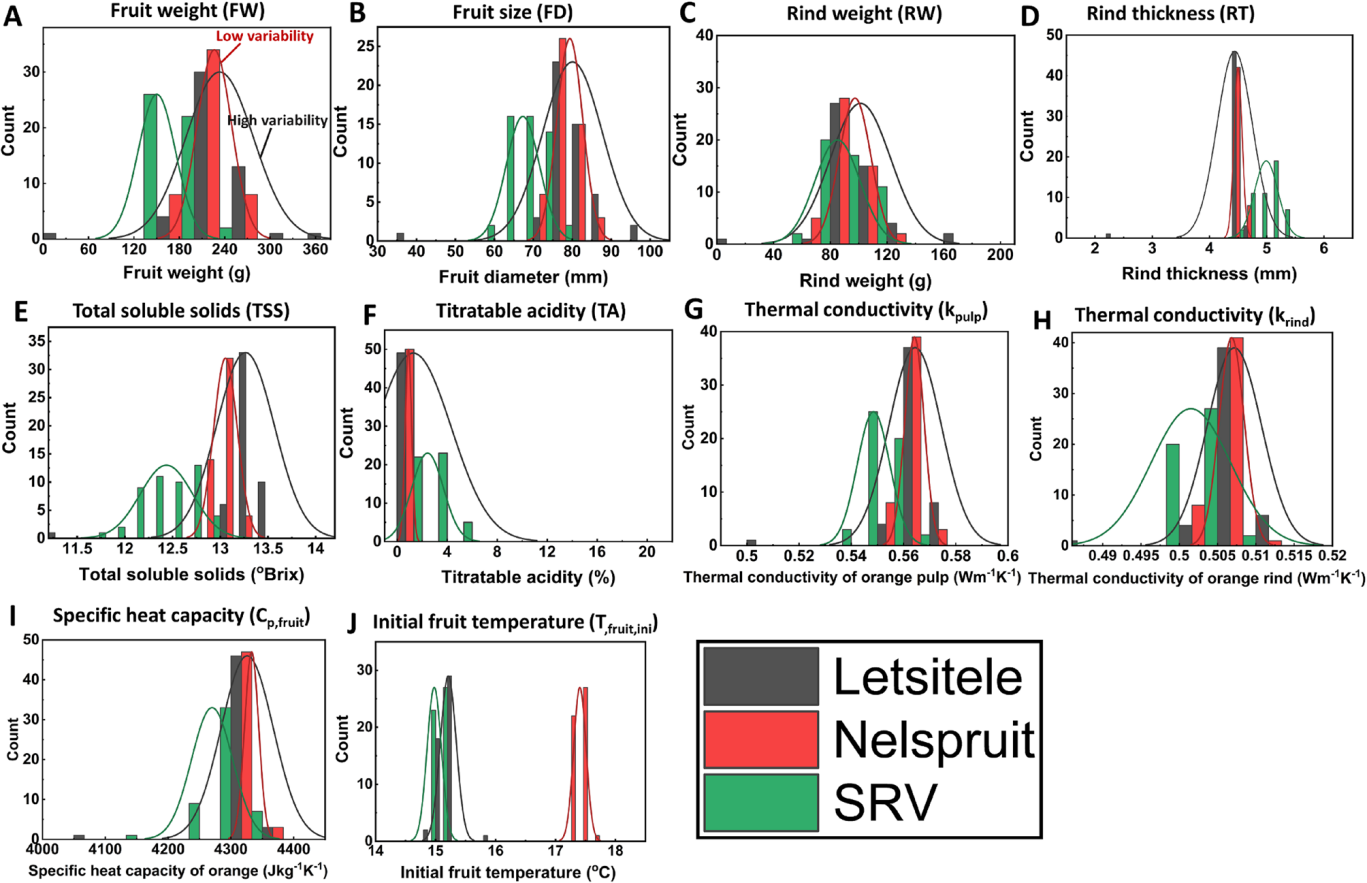
Variation in the physicochemical properties of oranges at harvest grown in Letsitele, Nelspruit, and Sunday’s River Valley (SRV) using a histogram with distribution plot for (**A**) fruit weight (FW, g), (**B**), fruit diameter (FD, mm), (**C**) rind weight (RW, g), (**D**) rind thickness (RT, mm), (**E**) total soluble solids (TSS, °Brix), (**F**) titratable acidity (TA, %), (**G**) thermal conductivity of orange pulp (k_pulp_, Wm^–1^ K^–1^), (**H**) thermal conductivity of orange rind (k_rind_, Wm^–1^ K^–1^), (**I**) specific heat capacity of orange (C_p,fruit,_ Jkg^–1^ K^–1^), and (**J**) initial fruit temperature (T_,fruit,ini_, °C). Note that at p < 0.05, the population means for each parameter are significantly different (For interpretation of the references to color in this figure legend, the reader is referred to the web version of this article).

Here, we analyze the variability of essential physicochemical properties of oranges at harvest from three regions in South Africa: Letsitele, Nelspruit, and SRV. The physicochemical parameters of interest include fruit weight (FW), fruit size (FD), rind weight (RW), rind thickness (RT), total soluble solids (TSS), titratable acidity (TA), thermal conductivity of orange pulp (k_pulp_), thermal conductivity of orange rind (k_rind_), specific heat capacity of the orange (C_p,fruit_), and initial fruit temperature (T_,fruit,ini_). These parameters are input parameters for our physics-based digital twins from orchard to retail. Figure 3 presents the distribution of the physicochemical properties of 'Valencia' oranges for 10 trees in 5 orchards each from Letsitele, Nelspruit, and SRV.

The results highlight statistically significant variations in the FW (Fig. 3A), FD (Fig. 3D), RW (Fig. 3C), RT (Fig. 3D), TSS (Fig. 3E), TA (Fig. 3F), k_pulp_ (Fig. 3G), k_rind_ (Fig. 3H), C_p,fruit_ (Fig. 3I), and T_,fruit,ini_ (Fig. 3J) of oranges at harvest between the growing regions (p < 0.05). Specifically, the variability of FW, FD, RW, RT, TA, k_pulp_, and C_p,fruit_ is highest for oranges produced in Letsitele, followed by SRV, while Nelspruit oranges exhibit the lowest variability for the same parameters. On the other hand, the variability of TSS and fruit temperature is highest for oranges from SRV compared to Letsitele and Nelspruit. The differences in these input parameters across different regions in South Africa result in the varying end quality of oranges at retail from these regions. This variability in the initial fruit properties can influence overall fruit quality, shelf life, the occurrence of chilling injury, as well as sugar and acidity levels of the oranges consumed by customers. Understanding and quantifying the impact of this variability is crucial for ensuring the delivery of high-quality oranges from each growing region to meet consumer expectations.

### How does the variability between growing regions impact the postharvest fruit quality at retail? (scenario 1)

**Figure 4:**
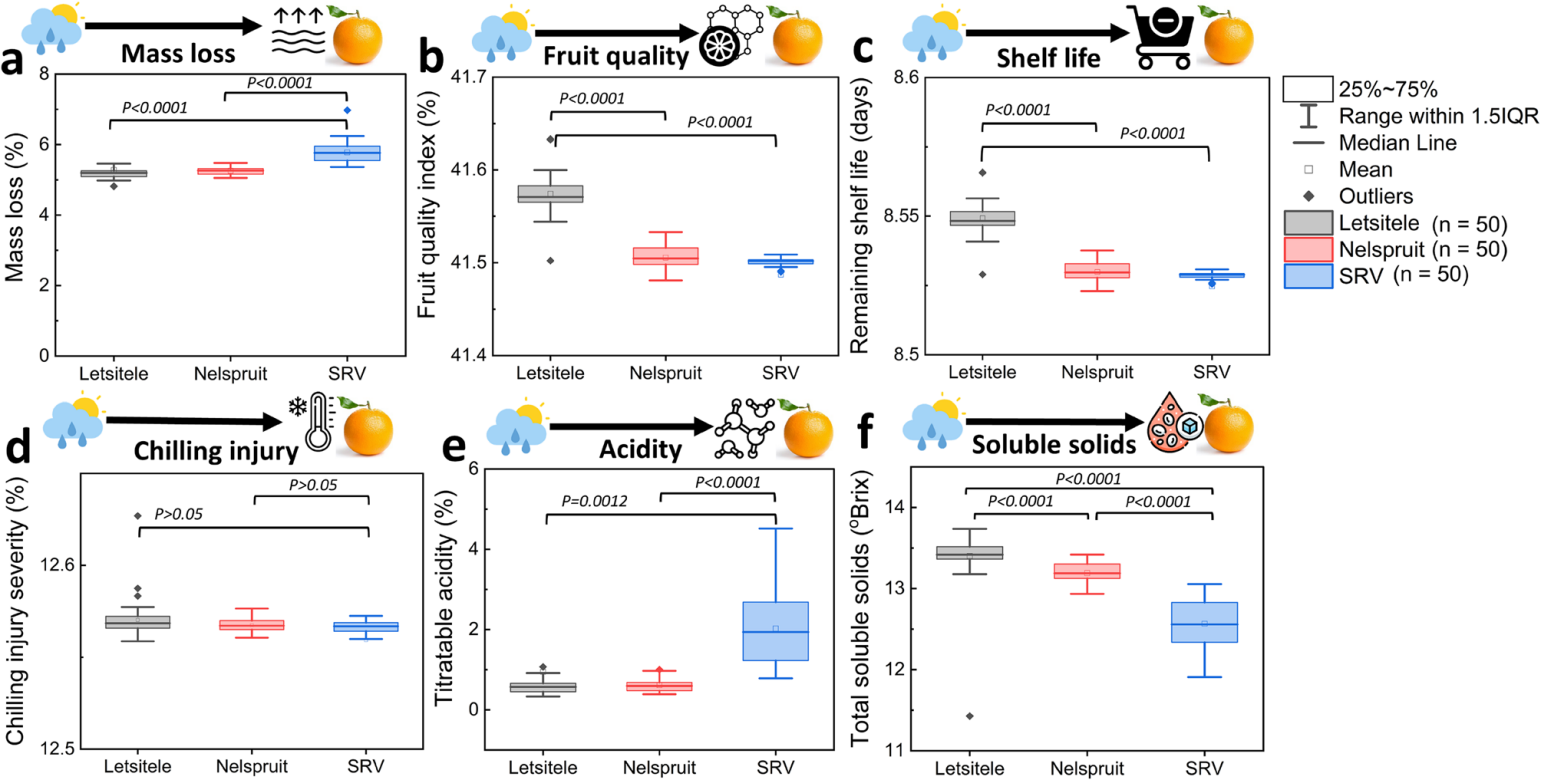
Impact of weather variability between Letsitele, Nelspruit, and SRV production regions on end fruit quality after long-time cold storage. (**a**) mass loss (%), (**b**) fruit quality index (FQI) (%), (**c**) remaining shelf life (RSL) (days), (**d**) chilling injury severity (%), (**e**) titratable acidity (TA) (%), and (**f**) total soluble solids (TSS) (°Brix). The boxplots represent the median (centre line), 75th upper and 25th lower quartiles (box limits), and 1.5 × the interquartile range (IQR, whiskers). p values were determined using Fisher test at a significance level of 0.05. Images for the figure icons are from Flaticon.com.

Here, we assess the impact of the differences in the growing conditions between Letsitele, Nelspruit, and SRV on mass loss, fruit quality index (FQI), remaining shelf life (RSL), chilling injury, TSS, and TA of oranges at retail using digital twin (Fig. 4). We do this without considering the hygrothermal differences in the local transportation between different growing regions. Figure 4a shows that the weather differences between Letsitele, Nelspruit, and SRV significantly impact the fruit mass loss at retail. Differences in the growing conditions between regions also significantly impact the fruit quality index (FQI) (Fig. 4b), remaining shelf life (RSL) days (Fig. 4c), chilling injury (Fig. 4d), titratable acidity (TA) (Fig. 4e) and total soluble solids (TSS) (Fig. 4f). This impact of the variability between growing regions on FQI and RSL at retail is the largest for oranges produced in SRV and Nelspruit compared to those of the hot semi-arid Letsitele. The FQI and RSL of oranges produced in Letsitele were 0.3% and 10% higher than oranges from SRV. The hot semi-arid growing region has a higher heat unit, indicating a faster growth rate than SVR. This results in higher fruit size and weight, increasing shelf life compared to smaller oranges^51^. Smaller citrus size has resulted in shorter shelf life after long cold storage^52^.

Interestingly, there is a significant difference between the FQI and RSL of oranges produced in Letsitele and those of Nelspruit, even though they have similar weather conditions during the growth process^40^. One reason for this disparity is the differences in the harvest fruit temperature between the two regions. Oranges harvested at lower temperatures (~ 14 °C for Letsitele) tend to have a longer shelf life compared to those harvested at higher temperatures (~ 17 °C for Nelspruit). The impact of the variations in weather growing conditions on mass loss (~ 10% more), TA (~ 1% higher), and TSS (~ 4% lower) at retail is most significant in the temperate oceanic climate of SRV compared to the hotter climate of Letsitele and Nelspruit. Fruit harvested from SRV are characterized by reduced fruit size and rind weight^21^. Small fruit size means the fruit has a more exposed surface area through which moisture can evaporate due to a larger surface area relative to their volume. As a result, fruit from SRV may experience higher mass loss during cold storage compared to larger oranges. Similar effects of fruit size on citrus mass loss during cold storage have been reported^52,53^.

Additionally, these oranges from SRV have high TA and lower TSS than those from hot, semi-arid growing climates. A higher TSS and lower TA in warmer citrus growing regions (e.g. Letsitele) have been reported in the literature^23,54^. More so, our digital twin showed no difference between the chilling injury severity of oranges produced in SRV and those from the warmer climates of Letsitele and Nelspruit. However, these findings contrast with the results from our controlled storage experiment, in which oranges from SRV had more chilling injuries compared to other regions^36^. One possible reason could be that our thermal damage model might not fully capture the complex interactions and dynamic conditions of actual fruit stored in a controlled cold atmosphere. Besides, we used actual hygrothermal citrus shipment data for our digital twin. Nonetheless, to reduce mass loss and maintain an optimal shelf life of fruit from the cooler climate of SRV, it is important to maintain the right temperature and humidity throughout the supply chain. For fruit from warmer climates, the energy demand during the cold chain can be reduced by increasing the temperature in the cold chain if the export protocol allows it. This is because fruit from this climate have a significantly longer shelf life but a risk of chilling injury after long cold storage than those from cooler climates.

Due to weather variability, we also see some end-quality variability between individual fruit from a specific growing region. The mass loss between individual oranges from SRV on arrival at the retail shows up to 0.4% variability and larger outliers compared to 0.2% for fruit harvested from Letsitele (Fig. 4a). This is significant considering that the average mass loss of the entire batch of oranges from Letsitele and SRV upon arrival at the retail is 5.3% and 5.6%, respectively. The FQI (Fig. 4b), RSL (Fig. 4c), and chilling injury (Fig. 4d) variability at retail between individual fruit from all growing regions are less than 0.05%, 0.05 days, and 0.05%, respectively. This means there is no significant difference in the final fruit quality, shelf life, or thermal damage of individual oranges from all regions upon arrival at retail. Figure 4e shows that the TA variability at retail between individual fruit from Letsitele and Nelspruit is up to 0.3% and ~ 2% for SRV. This means that more than 90% of the oranges from SRV contain different acidity levels at retail, since the mean is 2%. The TSS variability between individual fruit from SRV is ~ 1°Brix, while those of Letsitele and Nelspruit is 0.3°Brix (Fig. 4f). With a mean of 12.5°Brix, 8% of oranges from SRV will contain different degrees of sugar content at the retail. A similar impact of pre-harvest variability on mass loss and TSS of oranges at retail has also been reported^14^. These findings suggest that growers from a specific growing region should expect statistically significant differences in the fruit quality of their oranges. In general, optimizing harvest temperatures, particularly in warmer climates, managing temperature and humidity throughout the supply chain, and leveraging digital tools can significantly enhance the shelf life and quality consistency of oranges from different regions. Collaborative efforts among growers, suppliers, transporters, and retailers are essential to minimize quality loss and ensure high-value produce reaches consumers.

### How does the postharvest hygrothermal variability between growing regions affect fruit quality at retail? (scenario 2)

**Figure 5:**
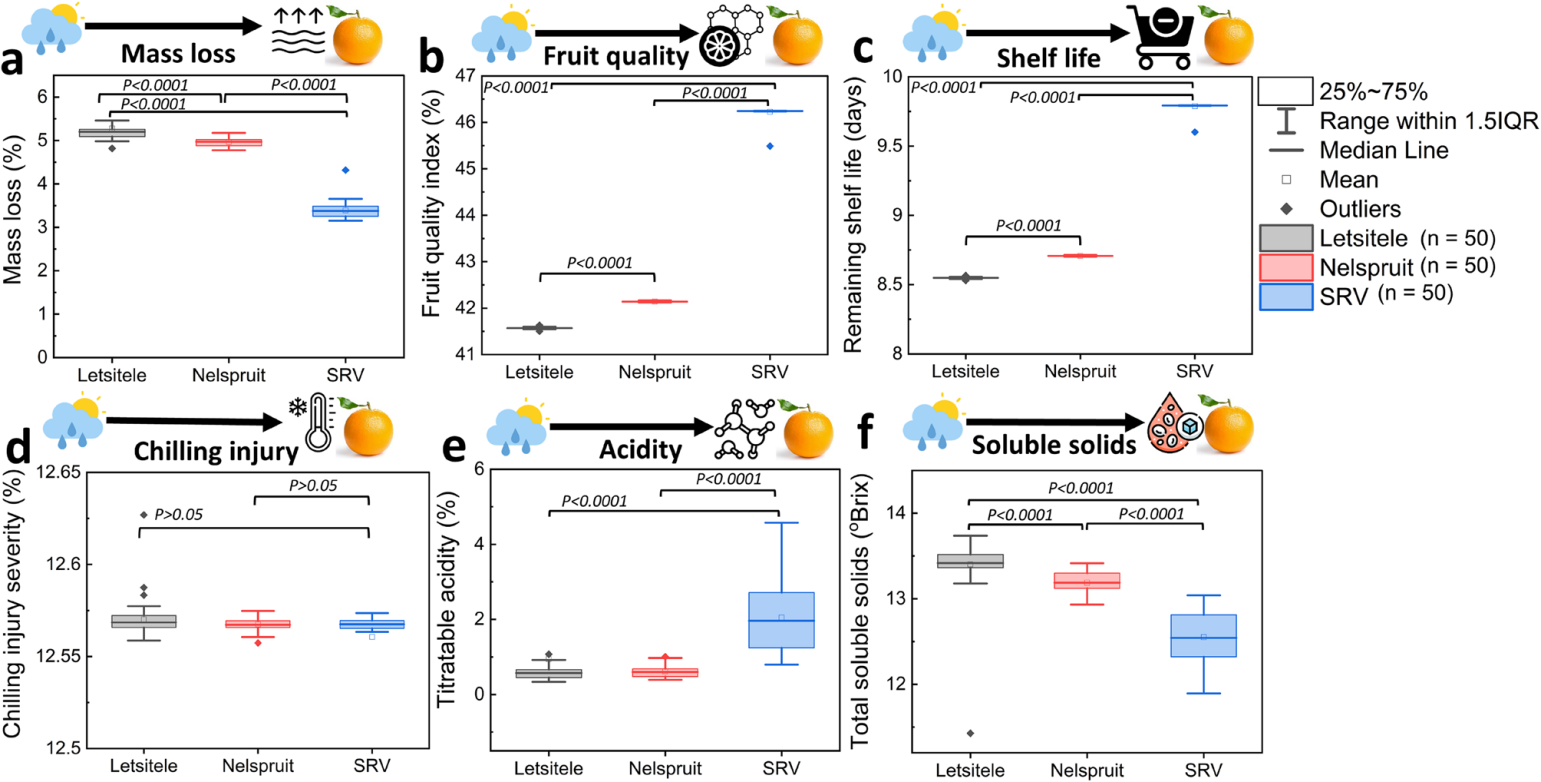
Impact of weather variability between growing areas on (**a**) mass loss (%), (**b**) fruit quality index (FQI) (%), (**c**) remaining shelf life (RSL) (days), (**d**) chilling injury severity (%), (**e**) titratable acidity (TA) (%), and (**f**) total soluble solids (TSS) (°Brix) considering postharvest hygrothermal differences. The boxplots represent the median (centre line), 75th upper and 25th lower quartiles (box limits), and 1.5 × the interquartile range (IQR, whiskers). p values were determined using Fisher test at a significant level of 0.05. Images for the figure icons are from Flaticon.com.

We quantify the impact of weather variability and the hygrothermal differences in the local transport chain between fruit from the three growing regions on retail end-quality (Fig. 5). This quality includes mass loss, fruit quality index (FQI), remaining shelf life (RSL), chilling injury, TSS, and TA of oranges. We simulated 50 oranges harvested from Letsitele, Nelspruit, and SRV going through three unique local supply chains from the different orchards to cold storage (Fig. 1B). We use the same cold chain for all oranges from a South African port to a Western European retailer. Similar to scenario 1, the hygrothermal variations during local transport lead to differences in the mass loss of oranges from growing regions when they reach retail (Fig. 5a). We also see a large variability in mass loss between individual fruit from a specific growing region. Fruit from SRV experienced lower mass loss postharvest compared with those from Letsitele and Nelspruit, contrary to the results from scenario 1. This is due to lower environmental temperature and higher humidity during the local unrefrigerated transportation of oranges from SRV compared to warmer atmospheric conditions of Letsitele and Nelspruit. These findings are consistent with the literature on postharvest variability and its effect on mass loss^14,55^.

Differences in the growing weather conditions have a large impact on FQI, RSL, TA, and TSS (Fig. 5b–c, e–f). The hygrothermal differences in the local supply chain significantly impact the FQI (Fig. 5b) and RSL (Fig. 5c) of oranges at retail. The FQI and RSL of fruit from SRV increased notably (FQI ~ 12%, RSL ~ 15%) compared to scenario 1 due to postharvest hygrothermal changes in the local supply chain. Similar to scenario 1, there was no significant difference in the chilling injury susceptibility across all the regions (Fig. 5d).

Furthemore, the hygrothermal variability during local transport did not have a significant impact on TA (Fig. 5e) and TSS (Fig. 5f), which followed the same trend as in scenario 1. This means that the impact of the variations in growing weather conditions on TA and TSS at retail remains most significant for oranges from temperate oceanic climate of SRV, compared to the hotter climates of Letsitele and Nelspruit. Oranges from SRV exhibited higher acidity levels and lower sugar content.

Overall, there is substantial variability in most fruit quality between individual fruit from a specific growing region, with SRV showing the largest impact, especially for TA and TSS. The trend for chilling injury, TA, and TSS remains similar to scenario 1, indicating that fluctuations in humidity and temperature during local transport did not significantly affect these parameters. However, hygrothermal disparities during unrefrigerated transport impacted mass loss, overall fruit quality, and remaining shelf life of oranges from different regions. These findings suggest that appropriate temperature and humidity levels during local transport need to be introduced, especially for fruit from cooler climates like SRV, to reduce mass loss and maintain quality. In addition, cold chain energy usage can be optimized by adjusting storage temperatures based on the origin of the fruit, particularly for fruit from warmer climates.

### Which weather parameters have the largest impact on postharvest fruit quality?

**Figure 6:**
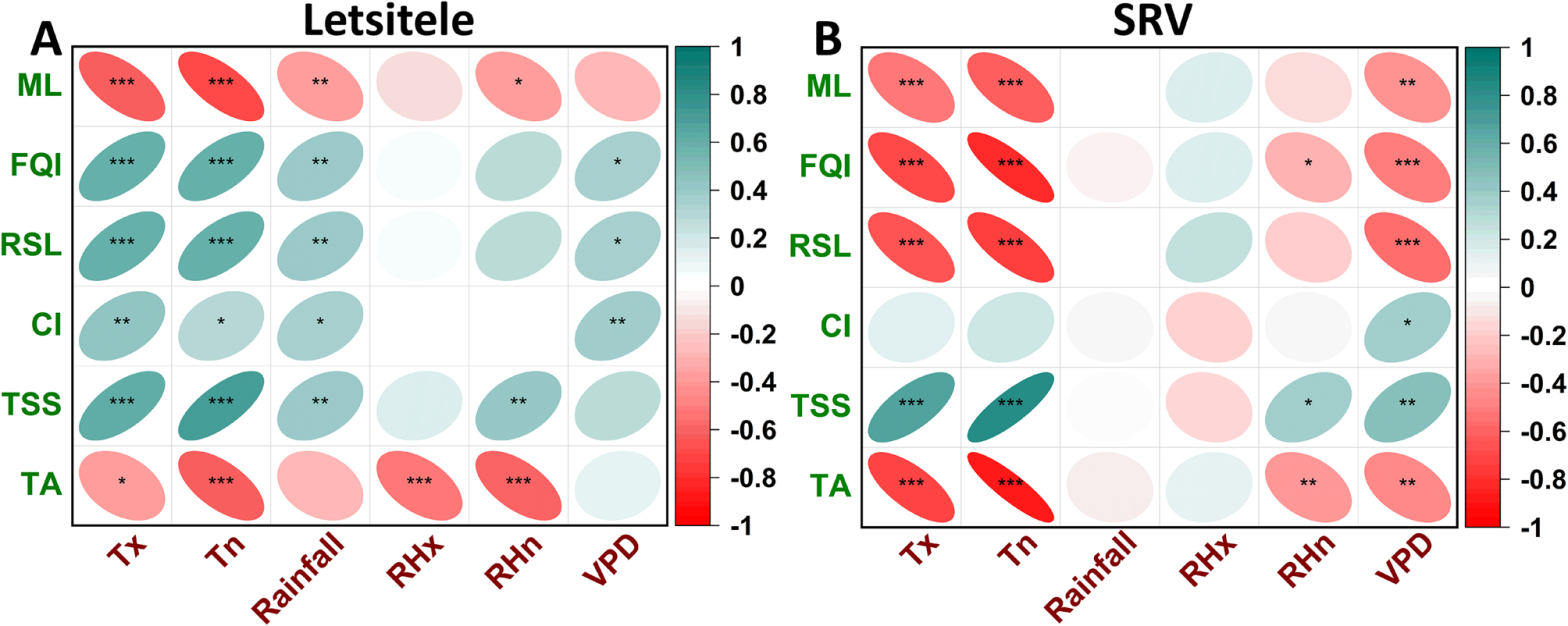
Pearson’s correlation coefficients of maximum temperature (Tx, °C), minimum temperature (Tn, °C), rainfall (mm), maximum relative humidity (RHx, %), minimum relative humidity (RHn, %), and vapor pressure deficit (VPD) (x-axis in red) on mass loss (ML, %), fruit quality index (FQI, %), remaining shelf life (RSL, days), chilling injury (CI, %), total soluble solids (TSS, °Brix), and titratable acidity (TA, %) of oranges (y-axis in green), for (**A**) Letsitele, and (**B**) Sunday’s River Valley (SRV) regions. The positive (green) and negative (red) correlations are indicated, and the color gradient and size of ellipse shape depict each correlation's strength. Black asterisks represent p-value as *** for p ≤ 0.001, ** for p ≤ 0.01, and * for p ≤ 0.05.

In a correlation plot, we show the relationship between environmental factors during fruit growth and various retail fruit quality attributes. Using this, we discuss the weather parameters that impact the postharvest fruit quality of oranges at retail, produced in warm and cool climates (see Fig. 6). The differences in weather conditions between growing regions, such as minimum temperature (Tn), maximum temperature (Tx), minimum relative humidity (RHn), maximum relative humidity (RHx), rainfall, and vapor pressure deficit (VPD), significantly impact the final postharvest quality of oranges.

For the hot semi-arid region of Letsitele (Fig. 6A), temperature (both minimum and maximum) is the most influential factor affecting various aspects of postharvest orange quality. The key quality parameters influenced by minimum and maximum temperatures include mass loss, fruit quality index (FQI), remaining shelf life (RSL), chilling injury, total soluble solids (TSS), and titratable acidity (TA). Temperature positively influences FQI, RSL, chilling injury, and TSS while negatively affecting mass loss and TA at retail. Rainfall is the next most significant factor, showing a strong correlation with mass loss, FQI, RSL, chilling injury, and TSS of oranges at retail. VPD positively influences FQI, RSL and chilling injury of oranges at retail. Minimum relative humidity (RHn) significantly affects mass loss, TSS, and TA, while maximum relative humidity (RHx) correlates with TA of oranges at retail.

For the coastal SRV region (Fig. 6B), both minimum (Tn) and maximum (Tx) temperatures are also primary factors strongly influencing mass loss, fruit quality index (FQI), remaining shelf life (RSL), total soluble solids (TSS), and titratable acidity (TA). However, in contrast to the warmer climate of Letsitele, these temperature parameters negatively affect FQI and RSL in the SRV region. Similarly, vapor pressure deficit (VPD) negatively impacts these quality parameters of oranges at retail, which is not observed in Letsitele.

The differences in the effects of temperature and VPD on orange quality and shelf life between Letsitele and coastal SRV are primarily due to the distinct climatic conditions, the adaptation of oranges to these conditions, and the interaction between temperature and VPD in each region. These differences highlight that temperature and VPD extremes, whether high or low, play a critical role in determining the postharvest quality of oranges produced in different regions. Furthermore, minimum relative humidity (RHn) moderately affects FQI, TSS, and TA of oranges at retail. This underscores the importance of considering regional climatic factors when evaluating the postharvest quality and shelf life of oranges.

Overall, these findings further show that warmer temperatures can affect fruit quality, making it essential to consider temperature variations during harvest planning to maintain optimal fruit quality. Managing harvest timing during rainy periods can help maintain fruit quality. These insights provide valuable guidelines for optimizing harvest planning and postharvest logistics, enabling growers to enhance the quality and shelf life of oranges through region-specific strategies.

## Conclusions

This study quantified the impact of the differences in the growing weather conditions between Letsitele, Nelspruit, and Sunday’s River Valley (SRV) regions of South Africa on the postharvest end-quality of oranges via a physics-based digital replica. We developed mechanistic digital twins of 50 Valencia oranges per growing region. These digital twins were coupled with measured real-world postharvest hygrothermal data and predicted fruit quality at retailers. Our study demonstrated that regional weather variations in physicochemical properties at harvest due to differences in growing temperature, humidity, rainfall, wind speed, and solar radiation, significantly impact the postharvest end-fruit quality of oranges from South Africa. These physicochemical parameters of fruit at harvest include fruit weight (FW), fruit size (FD), rind weight (RW), rind thickness (RT), total soluble solids (TSS), titratable acidity (TA), thermal conductivity of orange pulp (k_pulp_), thermal conductivity of orange rind (k_rind_), specific heat capacity of the orange (C_p,fruit_), and initial fruit temperature (T_fruit,ini_). The key conclusions derived from this study are as follows:

- There are statistically significant variations in the physicochemical properties of oranges at harvest between the growing regions.
- The weather variability between growing regions resulted in significant differences in the mass loss, FQI, RSL, TA, and TSS of oranges at retail. Retailers should be aware of these statistically significant differences in the quality of oranges from different growing regions. The impact of weather variations is most pronounced in the temperate oceanic climate of SRV compared to the hotter climates of Letsitele and Nelspruit.
- Oranges from SRV experience approximately 10% higher mass loss, ~ 10% lower remaining shelf life (RSL), 0.3% lower fruit quality index (FQI), 1% higher titratable acidity (TA), and 4% lower total soluble solids (TSS) at retail compared to oranges from Letsitele. This finding also indicates that smaller oranges are prone to higher mass loss and acidity, and have a shorter shelf life during postharvest storage due to their larger surface area relative to volume compared to larger oranges. By optimizing irrigation, nutrient, temperature, and soil management practices, growers in cooler climates can produce larger fruit, reduce mass loss, and enhance postharvest shelf life. Additionally, retailers could tailor marketing strategies and cold storage based on region-specific attributes of oranges. For instance, oranges from a certain could be promoted for their distinct taste profiles (e.g., based on Brix/acid ratio), while cold storage at the retail could be implemented to maintain quality.
- The hygrothermal variations in the local transport of oranges between different regions have a large impact on mass loss (up to ~ 2 times more), FQI (up to ~ 12% more), and RSL (up to ~ 15% more) at retail. These findings underscore the importance of optimizing transport conditions, especially for fruit from cooler climates like SRV, to reduce mass loss, increase shelf life and maintain quality.
- A large postharvest end-quality variability between individual fruit from a single growing region exists due to weather variability, with the temperate oceanic climate of SRV having the biggest impact.
- Oranges should be harvested at optimal temperatures to enhance shelf life. For example, lower harvest temperatures (around 14 °C), like in Letsitele, result in longer shelf life compared to higher temperatures (around 17 °C), as in Nelspruit. For oranges from warmer climates like Letsitele and Nelspruit, energy usage in the cold chain can be optimized by slightly increasing storage temperatures (considering phytosanitary standards), as these fruit have a lower risk of chilling injury and longer shelf life.

This study offers crucial insights into how regional weather differences affect fruit quality when it reaches the retail stage. These findings are significant for various stakeholders, including growers, retailers, logistic companies, researchers, policymakers, and other supply chain participants. They provide a foundation for informed decision-making, facilitating consistent production and consuming top-quality fruit. In practice, this research opens doors to innovative supply chain strategies. For instance, understanding how production climates impact fruit quality allows for better managing production practices and optimizing shipping schedules per supply region. By aligning shipments with higher ambient temperatures at the destination, substantial energy and cost savings can be achieved. These findings also serve as a call to all stakeholders—growers, suppliers, transporters, and retailers—to ensure a coherent strategy that minimizes quality loss, reduces waste, and enhances the overall value of the produce in the market.

## Data Availability

The pre-harvest digital twin model output used as the model input of our digital twin in the current study is available from the corresponding author upon request. All other data generated or analyzed during this study are included in this published article.

## Symbols

*ρ*_*i*_: Density of material (kg m^–3^)
*C*_pi_: Specific heat capacity of material (J kg^–1^ K^–1^)
*k*_i_: Thermal conductivity of the material (W m^–1^ K^–1^)
*Q*_resp,i_: Volumetric heat of respiration (W m^–3^)
$\nabla$: Spatial derivative operator (–)
$${T}_{i}$$: Fruit temperature (K)
*i*: Fruit pulp and rind domain
$n$: Unit vector normal to the surface (–)
*H*_c_: Convective heat transfer coefficient (W m^–2^ K^–1^)
*T*_air_: Delivery air temperature (K)
${k}_{air}$: Thermal conductivity of air (W m^–1^ K^–1^)
${C}_{p,fruit}$: Specific heat capacity of orange (J kg^–1^ K^–1^)
${k}_{pulp}$: Thermal conductivity of the orange pulp (W m^–1^ K^–1^)
${k}_{rind}$: Thermal conductivity of the orange rind (W m^–1^ K^–1^)
*A*_cross_: Cross-sectional area of the bottom of the cargo space (m^2^)
*U*_actual_: Actual airspeed around the orange fruit (m s^–1^)
*D*_fruit_: Diameter of the orange fruit (m)
r_pulp_: Radius of pulp (mm)
r_rind_: Radius of rind (mm)
rind_thick_: Rind thickness (mm)
*Nu*: Nusselt number (–)
*Re*: Reynolds number (–)
*ν*_air_: Kinematic viscosity of air (m^2^ s^–1^)
*δ*_wv__,air_: Coefficient of diffusion of water vapor in air (m^2^ s^–1^)
*Pr*: Prandtl number for air (–)
*µ*_air_: Absolute viscosity of air (kg m^–1^ s^–1^)
*µ*_air, rind_: Viscosity of air at the fruit surface (kg m^–1^ s^–1^)
*T*_*ini*_: Initial air temperature (°C)
*T*_*,fruit,ini*_: Initial fruit temperature (°C)
${k}_{t}$: Convection mass transfer coefficient (s m^–^^1^)
*P*_v,rind_: Surface/rind vapor pressure (Pa)
*P*_v,air_: Ambient vapor pressure (Pa)
*k*_rind_: Moisture migration through the rind (s m^–1^)
*k*_air,mass_: Air film mass transfer coefficient (s m^–1^)
*Sc*: Schmidt number (–)
*Sh*: Sherwood number (–)
${T}_{rind}$: Surface/rind temperature (K)
*T*: Temperature (K)
*C*_pair_: Specific heat capacity of air (J kg^–1^ K^–1^)
${RH}_{air}$: Relative humidity of the air (%)
RHx: Maximum relative humidity (%)
RHn: Minimum relative humidity (%)
${A}_{s}$: Surface area of the fruit (m^2^)
${FW}_{ini}$: Initial mass of the fruit (kg)
*k*_*i*_: Rate constant (s^–1^)
*n*_*i*_: Order of the reaction (–)
*A*_*i*_: Quality attributes (–)
E_a,i_: Activation energy (J mol^–1^)
R: Ideal gas constant (J mol^–1^ K^–1^)
rind_thick,0_: Initial rind thickness (mm)
r_pulp,0_: Initial pulp radius (mm)
*T*_*max*_: Daily maximum temperature (^o^C)
*T*_min_: Daily minimum temperature (^o^C)
*t*: Time (s)
*Ω*(t): Damage integral as a function of time (–)
*k*_0,ci_: Pre-exponential factor (s^–1^)
*E*_a,ci_: Activation energy for chilling injury (J mol^–1^)
Q_10_: Ratio of the rate constants at temperatures T and T + 10 K (= k_T+10_/k_T_) (–)
C: Integration constant
RH: Relative humidity (%)

## Abbreviations

VPD: Vapor pressure deficit
ARC: Agricultural Research Council
SA: South Africa
EPIC: Environmental Policy Integrated Climate
HUF: Heat unit factor
HUI: Heat unit index
FW: Fruit weight
FD: Fruit diameter
RW: Rind weight
RT: Rind thickness
TSS: Total soluble solids
TA: Titratable acidity
RP: Heat of respiration
IQR: Interquartile range
RSL: Remaining shelf life
CI: Chilling Injury
FQI: Fruit quality index
ML: Mass loss
SC: Supply chain
DC: Distribution center
SRV: Sunday’s River Valley
